# CtBP maintains cancer cell growth and metabolic homeostasis via regulating SIRT4

**DOI:** 10.1038/cddis.2014.587

**Published:** 2015-01-29

**Authors:** L Wang, H Zhou, Y Wang, G Cui, L-j Di

**Affiliations:** 1 University of Macau, Macau, SAR of People's Republic of China; 2 School of life Sciences, Anhui Medical University, Hefei, Anhui Province, People's Republic of China; 3 Institute of Chinese Medical Sciences, University of Macau, Macau, SAR of People's Republic of China; 4 Bioengineering department, Zunyi Medical college, Zhuhai, Guangdong Province, People's Republic of China

## Abstract

Cancer cells rely on glycolysis to maintain high levels of anabolism. However, the metabolism of glucose via glycolysis in cancer cells is frequently incomplete and results in the accumulation of acidic metabolites such as pyruvate and lactate. Thus, the cells have to develop strategies to alleviate the intracellular acidification and maintain the pH stability. We report here that glutamine consumption by cancer cells has an important role in releasing the acidification pressure associated with cancer cell growth. We found that the ammonia produced during glutaminolysis, a dominant glutamine metabolism pathway, is critical to resist the cytoplasmic acidification brought by the incomplete glycolysis. In addition, C-terminal-binding protein (CtBP) was found to have an essential role in promoting glutaminolysis by directly repressing the expression of SIRT4, a repressor of glutaminolysis by enzymatically modifying glutamate dehydrogenase in mitochondria, in cancer cells. The loss of CtBP in cancer cells resulted in the increased apoptosis due to intracellular acidification and the ablation of cancer cell metabolic homeostasis represented by decreased glutamine consumption, oxidative phosphorylation and ATP synthesis. Importantly, the immunohistochemistry staining showed that there was excessive expression of CtBP in tumor samples from breast cancer patients compared with surrounding non-tumor tissues, whereas SIRT4 expression in tumor tissues was abolished compared with the non-tumor tissues, suggesting CtBP-repressed SIRT4 expression contributes to the tumor growth. Therefore, our data suggest that the synergistically metabolism of glucose and glutamine in cancer cells contributes to both pH homeostasis and cell growth. At last, application of CtBP inhibitor induced the acidification and apoptosis of breast cancer cells and inhibited glutaminolysis in engrafted tumors, suggesting that CtBP can be potential therapeutic target of cancer treatment.

Cancer cells require carbon source that mainly exists in circulating plasma, such as glucose and glutamine, for ATP production and biosynthesis.^1^ Glucose metabolism in cancer cells is mainly through the glycolysis pathway, and several intermediates during glycolysis are used as substrates for subsequent branching biosynthetic pathways such as the pentose phosphorylation pathway and glycine–serine synthesis pathways and so on.^2^ The consequence of cancer cell-specific glycolysis is the accelerated glucose consumption and continuing supply of building blocks of amino acids, fatty acids and nucleotides.^3, 4, 5^

Glutamine is the most abundant amino acid in the plasma and was thought to be the nitrogen carrier as its most important role.^6, 7^ The growth of some cancer cells display as glutamine-dependent, but the required glutamine exceeds the obligated nitrogen supply, suggesting that glutamine has other functions in supporting cancer cell growth.^1^ For instance, cancer cells are able to sustain the tricarboxylic acid (TCA) cycle by providing the intermediates through a process called anaplerotic metabolism pathway.^8^ Through the deamination reaction, glutamine can be converted to glutamate and α-ketoglutarate (αKG), and subsequently enter into the TCA cycle. This pathway is also known as glutaminolysis and there are two enzymes catalyzing this process consecutively. The first enzyme is glutaminase (GLS), converting glutamine to glutamate, and the second enzyme is glutamate dehydrogenase (GDH), converting glutamate to αKG.^6^ Each enzymatic reaction releases one molecule of ammonia into mitochondria, which can diffuse to the cytoplasm and extracellular space and contribute to cell survival.^9^ GLS activity was already shown to correlate with tumor cell growth.^7^ Inhibition of GLS activity prevents oncogenic transformation and retards cell growth.^10, 11^ Recent studies also suggested that GDH is essential to support cancer cell growth by supplying the essential TCA intermediate αKG.^12, 13^

The C-terminal-binding proteins (CtBP1/2) are a dimeric family of proteins encoded by two analogous genes, CtBP1 and CtBP2, which have extensive roles in animal cell development.^14^ By forming either heterodimers or homodimers in the presence of nicotinamide adenine dinucleotide, CtBP is able to interact with gene-specific transcriptional factors and recruit several known epigenetic modifying enzymes such as LSD1, HDACs, G9a and so on to the target genes.^15, 16^ CtBP was found to directly repress the expression of several important tumor suppressor genes, and is involved in the epithelial to mesenchymal transition (EMT) during the cancer cell metastasis and other processes.^17, 18^ Extensive profiles of CtBP-target genes are identified recently in breast cancer cells, supporting that CtBP is an independent factor for tumor initiation, progression and metastasis by transcriptionally regulating genes related to stem cell pathways, genome stability, EMT and cancer cell metabolism.^19^

In the present study, we report a novel CtBP function in promoting glutaminolysis and maintaining the pH homeostasis, which are indispensable for the survival of breast cancer cells. We also show that SIRT4 is a target of CtBP and has negative correlation to CtBP in tumors. Further studies discovered that targeting CtBP results in the increased tumor cell apoptosis owing to the breakdown of pH homeostasis in engrafted tumors, suggesting that CtBP can be a potential therapeutic target for breast cancer treatment.

## Results

### CtBP is essential in supporting cell growth and maintaining the pH homeostasis during tumor cell growth

**Figure 1:**
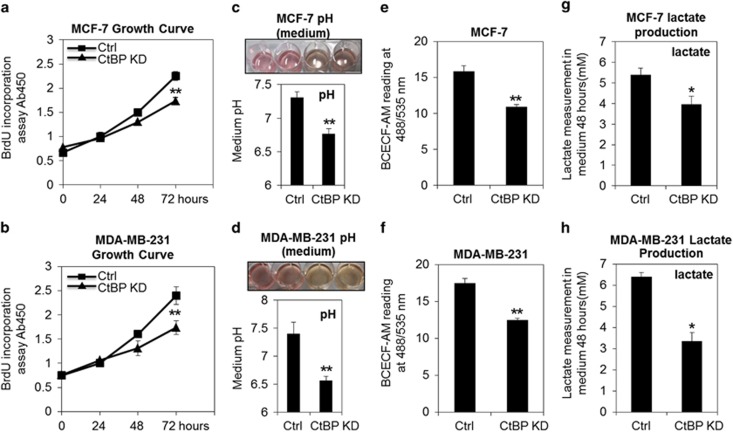
CtBP contributes to cell growth and regulates pH homeostasis in MCF-7 cells and MDA-MB-231 cells. (**a**) and (**b**) BrdU incorporation assay of growth curve in MCF-7 cells and MDA-MB-231 cells without (scramble siRNA-Ctrl) or with CtBP knockdown (CtBP KD). (**c** and **d**) The top pictures are the representative culture wells of both MCF-7 cells and MDA-MB-231 cells, the two left wells are transfected with scramble oligoes and the two right wells were transfected with CtBP knockdown oligoes, respectively. The bottom columns are the measured pH readings of the culture medium with or without CtBP knockdown. (**e** and **f**) The fluorescence signal of BCECF-AM is negatively correlated with intracellular acidity. So, the increased intracellular acidity is shown in both cell lines with or without CtBP knockdown. (**g** and **h**) Lactate production in both cell lines with or without CtBP knockdown. The error bars represent the S.D. of three independent replicates. *P<0.05, **P<0.01

To investigate the effect of CtBP on tumor cell growth, we performed CtBP knockdown in human mammary epithelial cancer cell lines MCF-7 cells and MDA-MB-231 cells. CtBP knockdown resulted in the significant retardation of cell proliferation indicated by BrdU incorporation assay in both cells (Figures 1a and b), and led to decreased Cyclin D1 and upregulated p21^waf1/cip1^ (Supplementary Figure 1), which indicate the decreased cell proliferation.^20, 21^ These data suggest that CtBP is essential to promote cancer cell growth.

Normally, growth retardation of cells is expected to delay the acidification of the medium because of the decreased glucose consumption and lactate production and secretion.^22^ Unexpectedly, the culture medium of CtBP knockdown cells exhibited an accelerated acidification in both MCF-7 cells and MDA-MB-231 cells compared with the control cells as indicated by the phenol-red indicator (Figures 1c and d). The medium pH dropped and cytoplasmic acidity increased significantly in both MCF-7 cells and MDA-MB-231 cells when CtBP was knocked down (Figures 1c–f). These results indicate that CtBP is essential to maintain the pH homeostasis of growing cancer cells. For actively proliferating cancer cells, release of lactate is increased because of the excessively increased anaerobic glycolysis, leading to decreased pH value that was also termed as 'Warburg effect'. However, CtBP knockdown decreases cell growth as well as the lactate production (Figures 1g and h), suggesting that lactate production is not the reason of the acidification of the culture medium.

### CtBP promotes cell growth and represses apoptosis of cancer cells via regulating glutaminolysis

**Figure 2:**
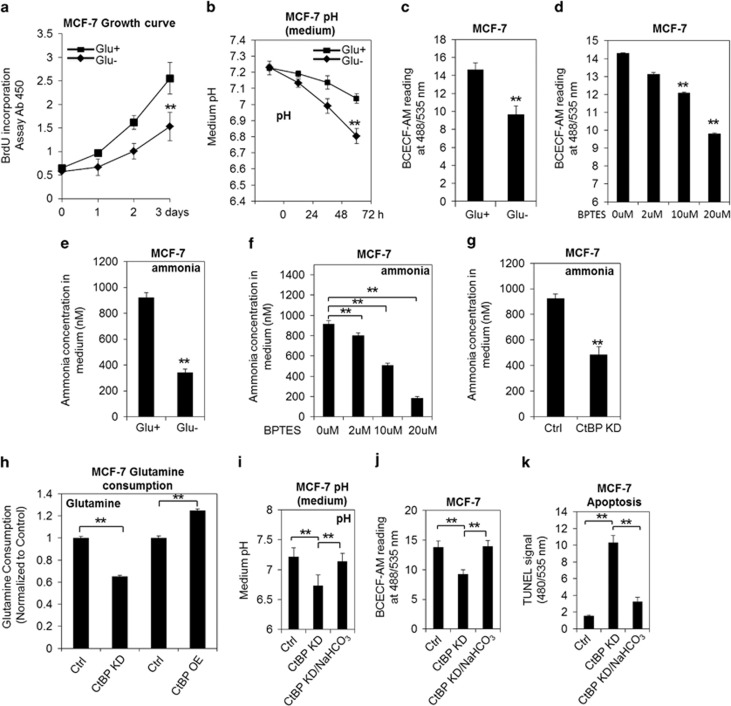
CtBP promotes cell growth and represses apoptosis via regulating glutaminolysis in cancer cells. (**a**) MCF-7 growth curves were shown when glutamine presence (Glu+) or absence (Glu-), indicated by BrdU incorporation assay. (**b**) Medium pH was monitored every 24 h for up to 72 h in MCF-7 cells with the conditions of glutamine presence and glutamine absence. (**c**) The increased intracellular acidity is shown in MCF-7 cells after culture for 48 h without the presence of glutamine. (**d**) Gradually increased intracellular acidity of MCF-7 cell after application of BPTES to the cells for 24 h at the indicated dosages is shown. (**e**) Ammonia production in MCF-7 cells with or without glutamine presence. (**f**) Ammonia production in MCF-7 cells after application of BPTES to the cells for 24 h at the indicated dosages. (**g**) Ammonia production in MCF-7 cells without or with CtBP knockdown (CtBP KD) for 72 h. (**h**) Glutamine consumption was determined in MCF-7 cells with CtBP knockdown (CtBP KD) or overexpression (CtBP OE). (**i**) Medium pH of MCF-7 cells with CtBP knockdown or CtBP knockdown plus NaHCO_3_. (**j**) The intracellular acidity is shown in MCF-7 cells with CtBP knockdown or with CtBP knockdown plus NaHCO_3_. (**k**) Apoptosis in MCF-7 cells upon CtBP knockdown was measured using TUNEL assay. The error bars represent the S.D. of three independent replicates. *P<0.05, **P<0.01

In addition to the glycolysis, recent studies suggested that incorporation of glutaminolysis into the TCA cycle might also contribute to the cancer cell growth.^23, 24, 25, 26^ In fact, when glutamine was withdrawn from the culture medium, the MCF-7 cells and the MDA-MB-231 cells showed significant decrease of proliferation (Figure 2a and Supplementary Figure 2A). Interestingly, the culture medium, as well as the cytoplasm, also exhibited accelerated acidification compared with the control cells when glutamine was withdrawn (Figures 2b and c, Supplementary Figures 2B and 2C). Bis-2-(5-phenylacetamido-1,3,4-thiadiazol-2-yl)ethyl sulfide (BPTES) is known to inhibit the glutaminolysis by inhibiting GLS and reducing the flux of glutamine to TCA cycle.^27^ Application of BPTES resulted in significant increasing of intracellular acidity (Figure 2d and Supplementary Figure 2D). A recent study suggested that glutamine may contribute to pH regulation by releasing ammonia.^28^ In fact, glutamine withdrawal directly affected the ammonia, releasing into the medium significantly (Figure 2e). BPTES treatment of MCF-7 and MDA-MB-231 cells resulted in reduction of ammonia (Figure 2f and Supplementary Figure 2E), suggesting that glutaminolysis-dependent ammonia production is critical to pH stability in cancer cells. Surprisingly, there was a significant decrease of ammonia level in the culture medium of both MCF-7 cells and MDA-MB-231 cells with CtBP knockdown (Figure 2g and Supplementary Figure 2F), suggesting that CtBP status influences intracellular pH via regulating ammonia production.

To confirm that CtBP regulates ammonia production by affecting glutaminolysis pathway, the glutamine consumption was measured in MCF-7 cells and MDA-MB-231 cells with CtBP knockdown or CtBP overexpression. CtBP knockdown significantly decreased the glutamine consumption, whereas CtBP overexpression increased the glutamine consumption (Figure 2h and Supplementary Figure 2G). Consequently, CtBP knockdown induced significant acidification of MCF-7 cells (Figures 2i and j), which is independent of reactive oxygen species (ROS) (Supplementary Figure 2H). CtBP knockdown also induced apoptosis judged by terminal deoxynucleotidyltransferase-mediated dUTP nick end labeling (TUNEL) assay (Figure 2k). Extra NaHCO3 supplementation recovered the pH, and importantly, rescued the cancer cell induced by CtBP knockdown (Figures 2i–k, Supplementary Figures 2I and 2J). These data strongly imply that CtBP positively facilitates glutaminolysis to release more ammonia and contributes to the maintenance of the metabolic homeostasis of cancer cells, which is crucial to protect the cells against acidification induced apoptosis.

### CtBP negatively regulates SIRT4 to affect glutaminolysis

**Figure 3:**
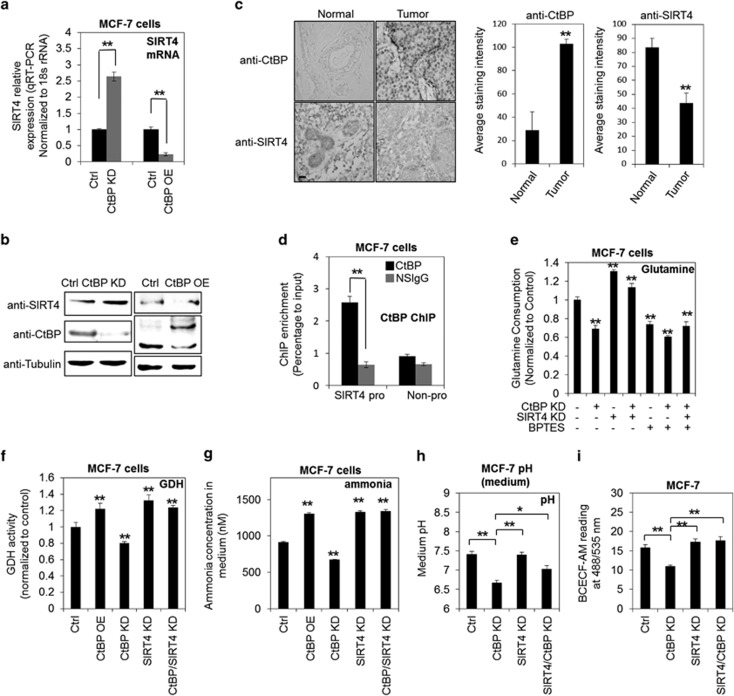
CtBP regulates glutaminolysis via repressing SIRT4. (**a** and **b**) SIRT4 expression was determined in MCF-7 cells with CtBP knockdown (CtBP KD) or CtBP overexpression (CtBP OE) by RT-PCR and western blotting. CtBP was overexpressed as fusion protein with GFP tag. (**c**) Left, representative immunohistochemistry (IHC) staining for CtBP and SIRT4 in human breast normal tissues and breast tumor tissues (scale bar: 25 um); middle and right, histograms to show CtBP and SIRT4 staining in both normal tissues and breast tumor tissues. The columns represent the average staining intensity by each antibody in IHC assays. (**d**) ChIP assay of CtBP binding at SIRT4 promoter. A neighbor region of SIRT4 gene without transcripts (Non-pro) was used as negative binding control region, and nonspecific IGG (NSIgG) was used as negative control for chromatin pull down. The binding was shown as percentage to input. (**e**) Glutamine consumption was determined in MCF-7 cells with different treatments as indicated. The measurements were performed after 72 h for gene knockdown and the BPTES dosage was 10 uM. (**f**) GDH activity was measured in cells treated by the conditions as indicated. The measurements were performed 72 h after treatment. (**g**) Ammonia production in response to the conditions as indicated in MCF-7 cells. (**h** and **i**) The measurement of both the medium pH and intracellular acidity of MCF-7 cells with conditions as indicated. The error bars represent the S.D. of three independent replicates. *P<0.05, **P<0.01. In (**e**, **f** and **g**) the *P-*values were calculated between the control sample and the indicated sample, respectively

SIRT4 is a known repressor of GDH and is located in the mitochondria.^23, 25, 26, 29^ Several recent studies demonstrated that SIRT4 is required to inhibit glutamine-dependent metabolism in response to DNA-damage stress and prohibit the tumor growth in different tissues.^23, 25, 26, 29^ Interestingly, in previous gene expression microarray data, SIRT4 mRNA showed significant upregulation upon CtBP knockdown in MCF-7 cells.^19^ In addition, a significant binding peak was identified at SIRT4 gene promoter in CtBP genome-wide-binding profile analysis (Supplementary Figure 3A). Consistently, CtBP was also found to have a significant binding peak at SIRT4 promoter in Encyclopedia of DNA Elements project (Supplementary Figure 3B).^30^ To validate these previous data, the SIRT4 expression was examined in MCF-7 cells and MDA-MB-231 cells. Knockdown of CtBP increased SIRT4 expression at both mRNA and protein level in MCF-7 cells and MDA-MB-231 cells, whereas CtBP overexpression downregulated SIRT4 expression significantly (Figures 3a and b,Supplementary Figures 3C and 3D). Next, we determined the expression pattern of SIRT4 and CtBP in human breast tumor samples. The immunohistochemistry staining showed that there was significant excessive expression of CtBP in tumor samples compared with surrounding non-tumor tissues, whereas SIRT4 expression in tumor tissues was abolished compared with the non-tumor tissues (Figure 3c). Such an inverse expression pattern of these two proteins can be confirmed by Pearson correlation analysis (R=−0.5908) and further reflects that CtBP negatively regulates SIRT4 expression in human breast tumor tissues (Supplementary Figure 3E). Furthermore, the excessive expression of CtBP in breast tumor samples highly suggests that CtBP is positively associated with breast tumor development. Then, chromatin immunoprecipitation (ChIP) assay demonstrated the binding of CtBP at SIRT4 gene promoter (Figure 3d and Supplementary Figure 3F), suggesting a direct regulation of SIRT4 by CtBP. In addition, SIRT4 knockdown, when combined with CtBP knockdown, was able to reverse the decreased glutamine consumption observed in CtBP knockdown cells (Figure 3e and Supplementary Figure 3G). As expected, both CtBP status and SIRT4 status no longer affect the reduced glutamine consumption rate upon BPTES treatment (Figure 3e and Supplementary Figure 3G), consistent to the inhibitory specificity of BPTES on GLS that catalyzes the glutaminolysis reaction one step earlier than GDH. To further confirm that CtBP affects glutamine consumption by influencing GDH activity, the enzymatic activity of GDH were measured. The data show that CtBP positively regulates GDH activity in a SIRT4-dependent manner because SIRT4 knockdown and CtBP knockdown combination reversed the decreased GDH activity observed in cells with CtBP knockdown alone (Figure 3f and Supplementary Figure 3H). Ammonia production is also regulated by CtBP in a SIRT4-dependent manner (Figure 3g and Supplementary Figure 3I). Consistently, the increased acidification upon CtBP knockdown was overcome when SIRT4 was knocked down together (Figures 3h and i, and Supplementary Figures 3J and 3K). Taken together, these findings highlight the role of CtBP in regulating glutaminolysis via repressing SIRT4 expression.

### CtBP-promoted glutaminolysis is essential to maintain cellular respiration activity

**Figure 4:**
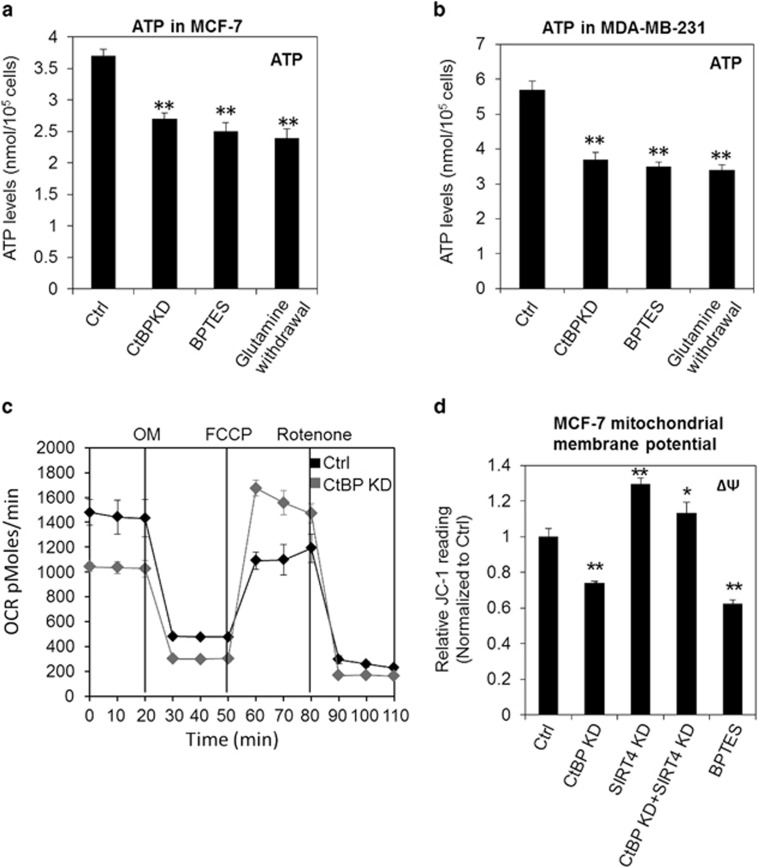
CtBP is essential to maintain the respiration activity. (**a** and **b**) ATP levels were measured in MCF-7 cells and MDA-MB-231 cells treated by CtBP knockdown (CtBP KD), BPTES and glutamine withdrawal. (**c**) OCR measurements of MCF-7 cells with or without CtBP. (**d**) Mitochondria membrane potential measurements in MCF-7 cells with conditions as indicated. The error bars represent the S.D. of three independent replicates. *P<0.05, **P<0.01. In (**a**, **b** and **d**) the *P-*values were calculated between the control sample and the indicated sample, respectively

Glutamine is essential for the growth of some cancer cells such as MCF-7 and MDA-MB-231 cells. Glutamine mainly serves as anaplerotic substance in supporting TCA cycle in cancer cells. In view of the critical role of CtBP in regulating glutaminolysis pathway and its positive influence on cancer cell growth via regulating SIRT4, we wonder if CtBP-regulated glutaminolysis pathway has global effect on cell metabolism. A significant decrease of ATP synthesis was observed when CtBP was knocked down in MCF-7 cells and MDA-MB-231 cells (Figures 4a and b). Similarly, decrease of ATP production can be seen in the cells treated by BPTES or cultured in glutamine-free medium (Figures 4a and b). Also, the oxygen consumption rate (OCR) decreased markedly in CtBP knockdown cells (Figure 4c). So we further measured the membrane potential ΔΨ of mitochondria,^31^ which directly correlates with the oxygen consumption and ATP production. BPTES treatment results in mitochondrial membrane potential decreased significantly as assessed by JC-1 staining of inter-membrane protons (Figure 4d), consistent to Weinberg *et al.*'s^32^ observation. CtBP knockdown resulted in the decreasing of mitochondrial membrane potential significantly, and SIRT4 knockdown increased the mitochondrial membrane potential slightly but significantly (Figure 4d). However, SIRT4 knockdown and CtBP knockdown together restored decreased mitochondrial membrane potential (Figure 4d), suggesting that the glutaminolysis is essential to maintain the proper function of mitochondria in cancer cells, which is regulated by CtBP and SIRT4.

### The inhibition of CtBP repressive activity by MTOB interrupts metabolic homeostasis and glutaminolysis in cancer cells

**Figure 5:**
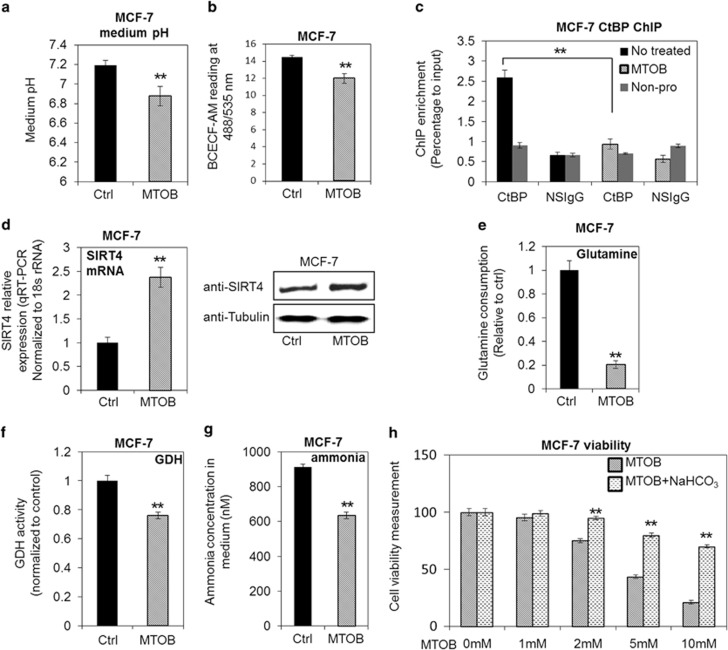
MTOB destroys the metabolic homeostasis *in vitro* in MCF-7 cells by inhibiting CtBP. (**a** and **b**) Both medium pH and intracellular acidity were measured in MCF-7 cells with or without MTOB treatment (10 mM) for 24 h. (**c**) Reduced CtBP binding at SIRT4 promoter was determined by ChIP assay in MCF-7 cells after the cells were treated by MTOB for 24 h. A non-relevant neighbor sequence of SIRT4 gene was used as negative control region at the binding assay and nonspecific IGG (NSIgG) was used for negative control in pull down. (**d**) Real-time PCR and western blotting measurement of SIRT4 gene expression in MCF-7 cells with or without MTOB treatment. (**e**) Glutamine consumption was measured in MCF-7 cells with or without MTOB treatment. (**f**) GDH activities of MCF-7 cells were measured with or without MTOB treatment. (**g**) Ammonia was measured in MCF-7 cells with or without MTOB treatment. (**h**) MTT assay of MCF-7 viability upon treatment by MTOB or MTOB plus extra NaHCO_3_ (3.7 g/l). The error bars represent the S.D. of three independent replicates. *P<0.05, **P<0.01

MTOB (4-methylthio-2-oxobutanoate) is a proven inhibitor of CtBP function with observed effect in inhibiting the engrafted tumor growth.^33^ Importantly, MTOB was shown to directly inhibit the repressive function of CtBP.^19, 33, 34^ Thus, the MTOB function in affecting the intracellular metabolic homeostasis was investigated. Application of MTOB accelerated the acidification of both culture medium and cytoplasm in MCF-7 cells (Figures 5a and b), indicating MTOB is capable of destroying the pH homeostasis of cancer cells. MTOB also accelerated the intracellular acidification of MDA-MB-231 cells significantly (Supplementary Figure 4A). To confirm that MTOB effect on pH is via CtBP and SIRT4, we performed ChIP assay and found that MTOB effectively alleviated CtBP binding at SIRT4 promoter (Figure 5c and Supplementary Figure 4B). The expression of SIRT4 increased as expected after MTOB treatment (Figure 5d and Supplementary Figure 4C). Consistently, the cells showed robust decrease in glutamine consumption in response to MTOB treatment (Figure 5e and Supplementary Figure 4D). The GDH activity, as well as the ammonia production, were also significantly inhibited by MTOB (Figures 5f and g and Supplementary Figures 4E and 4F). In order to evaluate the potential of CtBP to be breast tumor therapeutic target, the cell viability was measured and we observed that MTOB was able to induce significant decrease of cell viability, which is consistent with Straza's observation in HCT116 cells.^33^ However, adding extra NaHCO3 together with MTOB significantly rescued the cell viability (Figure 5h and Supplementary Figure 4G), suggesting that pH homeostasis disturbance significantly contributes to MTOB-induced cell apoptosis. Collectively, the data indicate that chemicals targeting CtBP may be developed and contribute to cancer cell death and potentially can be used as therapeutic drug.

### MTOB induces cell apoptosis in engrafted tumors via regulating SIRT4 and GDH activity

**Figure 6:**
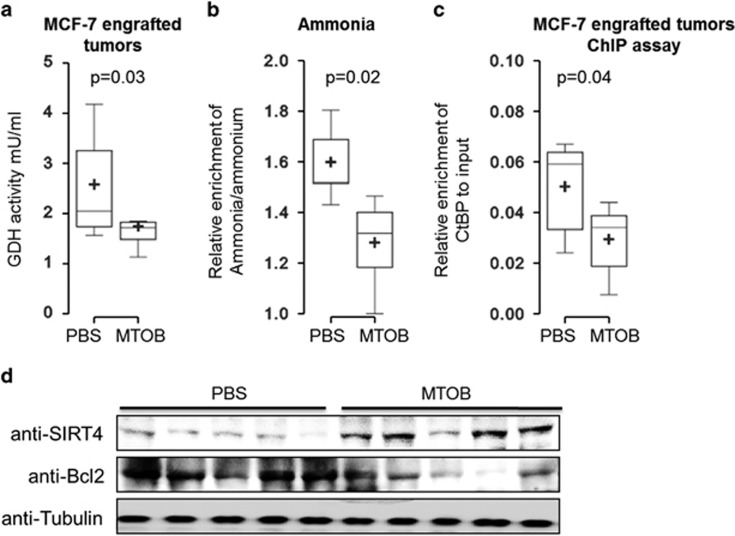
MTOB inhibits GDH activity and induces cell apoptosis in engrafted tumors. (**a** and **b**) GDH activity and ammonia production were determined in the engrafted tumors in mice treated by either PBS or MTOB (750 mg/500 g body weight) (*n*=6). (**c**) ChIP assay of CtBP enrichment at SIRT4 gene promoter in engrafted tumors from PBS or MTOB-treated mice. (**d**) Western blotting shows the SIRT4 level, BCL-2 level in tumors isolated from PBS or MTOB-treated mice and tubulin is the loading control

Then, the effect of MTOB on engrafted tumors was tested. Around 1 × 10^4^ MCF-7 cells were subcutaneously injected to immunodeficient nude mice to generate engrafted tumors. Then MTOB was applied to the tumors every 2 days for three times. The GDH activity of the tumor cells was measured. MTOB significantly decreased the GDH activity in engrafted tumors (Figure 6a). Accordingly, the ammonia level extracted from the MTOB-treated tumors was significantly lower than PBS-treated tumors (Figure 6b). The increased SIRT4 expression and the abolishment of CtBP binding at SIRT4 promoter in MTOB-treated tumors were also confirmed (Figures 6c and d, and Supplementary Figure 5), suggesting that MTOB inhibits CtBP activity to upregulate SIRT4 expression, which contributes to the repression of GDH activity and glutaminolysis activity *in vivo*. Bcl-2 is an antiapoptosis gene and its downregulation associates with increased apoptosis in MCF-7 cells. A direct consequence of MTOB-inhibited GDH activity decreased Bcl-2 level, which indicates the increased tumor cell apoptosis (Figure 6d). As long-term treatment of engrafted tumors by MTOB shrunk the tumors,^33^ these data suggest that the apoptosis induced by breakdown of the intracellular glutaminolysis pathway may be the reason of the MTOB-induced tumor shrinking. Together, these *in vitro* and *in vivo* data strongly suggest that targeting CtBP, and breaking the pH homeostasis of cancer cells, are feasible to treat breast cancer.

## Discussion

### pH homeostasis and cancer

**Figure 7:**
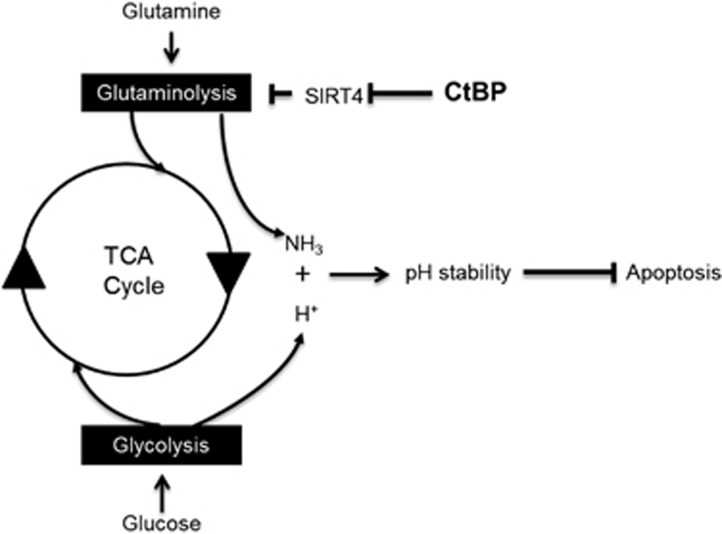
Diagram to show the interaction between glycolysis and glutaminolysis mediated by CtBP and SIRT4 in cancer cells. Both glucose and glutamine are independent nutrients for cancer cell growth. Glucose is metabolized through the glycolysis pathway and glutamine is metabolized through the glutaminolysis pathway. However, the metabolism of glucose in cancer cells is not complete and may lead to the accumulation of excessive acidic molecules and threaten the survival of cancer cells. Simultaneously, cancer cells also rely on glutamine as important carbon and nitrogen source. The by-product of glutaminolysis is ammonia which has an important role in neutralizing the glycolysis-associated acidification. CtBP promotes the glutaminolysis by directly regulating SIRT4 gene expression, which is a mitochondria repressor of glutaminolysis. Therefore, CtBP generates at least two kinds of benefit to cancer cell growth: promoting the production of ammonia that is essential to neutralize the acidification associated with incomplete glycolysis and increasing the glutamine supply, which is critical to support cancer cell proliferation

Several mechanisms are responsible for maintaining the pH within certain range and failure to retain the pH stability results in the apoptosis of cells.^35, 36, 37^ Surprisingly, our findings indicate a previous unrecognized mechanism through which the intracellular pH of cancer cells is regulated in favor of the cell growth (Figure 7). This mechanism relies on the excessive glutaminolysis pathway, which continually produces ammonia within the mitochondrial matrix in cancer cells. Actually, ammonia released in renal tubule epithelial cells, produced from glutaminolysis, is critical to form ammonium ions that is further metabolized to urea and excreted. This process is well known to be critical to maintain the cytoplasmic pH physiologically.^38^ In tumors, it was known that glutamine is mainly utilized as important carbon and nitrogen source. Our data, however, suggest that CtBP-regulated glutamine consumption in cancer cells forms an intracellular pH maintenance system and is critical to provide ammonia to balance the excessive acidification associated with tumor cell proliferation. According to our data, this glutamine-dependent pH management system is independent of other known mechanisms such as the cell membrane-bound proton exporters. Our findings of cancer cell dependence on glutaminolysis add an extra, but more general, strategy in dealing with the acidosis challenge, facing by the tumor cells.

### CtBP and cell metabolism

CtBP is much better known for its response to cell metabolism status than its impact on cell metabolism pathways.^19, 39, 40, 41, 42, 43^ Our finding that CtBP regulates glutamine metabolism in cancer cells, to our knowledge, is the first report demonstrating that the cancer cell proliferation promoting function of CtBP requires its involvement in the metabolic control. Because excessive expression of CtBP was observed in many different types of cancers, and associated with the more aggressive subtype of cancer patients with worse outcome, our identification of CtBP function in regulating cancer cell metabolic pathways and resultant intracellular pH homeostasis accounts for above observation and reveals insights into CtBP effect on the molecular mechanisms of cancer cell growth and points to the therapeutic potential of CtBP in cancer treatment. In addition, our findings may be meaningful to understand the important role of CtBP in the development of the related tissues as well. According to previous studies, CtBP is involved in repressing the differentiation and maintaining the proliferation of progenitor cells from multiple tissues.^41, 44, 45^ Thus, CtBP may be critical to maintain the proliferation of the progenitor cells in different tissues by regulating the cellular metabolic pathways. Particularly for those progenitor cells reside in hypoxic niches, glycolytic and anaerobic respiration is a preferred energetic pathway and CtBP could be the essential regulator to support their self-renewal and pH homeostasis.

### Glutamine and mitochondrial activity

Glutamine is the most abundant amino acid in the plasma. After entering into the cells, glutamine can be converted to glutamate, which is a versatile metabolic intermediate that connects with a wide variety of distinct biological processes such as synthesis of the antioxidant glutathione, amino-acid catabolism through transamination, anaplerotic supplementation for TCA cycle and so on.^6, 9, 11, 23, 24, 29^ Our observations reported here actually suggested that glutamine is also capable of promoting the oxidative phosphorylation and increases the mitochondrial membrane potential probably by providing more four carbon substances once it enters TCA cycle, which contributes to cancer cell growth. For cancer cells, there are at least several benefits can be obtained through the increased glutaminolysis. First of all, glutamine is important carbon and nitrogen source and can be supportive for tumor growth;^6, 7, 10, 12, 13, 25^ second, the by-product of glutaminolysis, ammonia, is important to suppress the acidosis pressure associated with the glycolytic proliferation;^9, 28, 38^ third, glutamine is also beneficial to suppress the ROS crisis in cancer cells;^32, 46, 47^ and at last, glutaminolysis is also contributive to increase the mitochondrial activity and enhances the ATP production.^29, 32^ Thus, our findings of CtBP-promoted glutaminolysis is an indispensable pathway for tumor cell survival and growth. In addition, we also observed that a possible tumor repressor, SIRT4, was demonstrated to negatively mediate CtBP-regulated glutaminolysis. As SIRT4-repressed glutaminolysis inhibited cancer cell growth,^25, 26^ our elucidation that SIRT4 acts as a target gene of CtBP further strongly supports the view that CtBP can be a potential target of tumor therapeutic strategy.

## Materials and Methods

### Chemicals and reagents

BPTES, 4-methylthio-2-oxobutyric acid (MTOB), are purchased from Sigma-Aldrich (St. Louis, MO, USA). BPTES was dissolved in DMSO at 5 mM as stock. MTOB was dissolved in medium to 250 mM and diluted to 10 mM final concentration in cell culture. The antibody to CtBP used for ChIP was purchased from Santa Cruz Biotechnology (Dallas, TX, USA) and is cross-reactive with both CtBP1 and CtBP2. The lactate, glutamine, glucose and ammonia colorimetric assay kits are purchased from Biovision (San Francisco, CA, USA) and Bioassay system (Hayward, CA, USA).

### Cell culture

MCF-7 cells and MDA-MB-231 cells were maintained in regular DMEM supplemented with 10% (v/v) FBS, penicillin-streptomycin (Invitrogen, now part of Thermo Fisher Scientific, Waltham, MA, USA) and insulin. For knockdown experiments, the procedure and oligo for CtBP knockdown is the same as described before.^19^ SIRT4 knockdown oligoes were purchased from Santa Cruz as mixed oligoes pool, and the procedure followed the manual provided by the manufacture. For overexpression experiments, CtBP expression vector was transfected into the indicated cells as described.^19^

### Cell growth curve measurement

The cell growth was measured using BrdU incorporation kit from CellSignal (Danvers, MA, USA) and followed the manufacture's procedure or MTT assay.

### RT-PCR and western blotting

Both experiments are performed following the standard protocol (see Supplementary data).

### ChIP

All ChIP experiments were carried out as described.^19^

### pH measurement

For culture medium, the medium was removed from the culture dish immediately after the dishes leaving the incubator, and the pH was measured using regular lab oratory pH meter. For measurement of cytoplasmic pH, a fluorescence probe BCECF-AM was applied. The fluorescence signal of BCECF-AM is positively correlated with intracellular pH and negatively correlated with intracellular acidity. BCECF-AM stock (5 mM) was purchased from Beyotime (Nantong, Jiangsu, China) and 10 uM final concentration was applied to the cells. Incubate the cells with BCECF-AM for 30 min. Wash away the extra BCECF-AM solution from the cells by PBS for three times. Then the cells are kept in PBS and the signals are read using the fluorescent plate reader with the excitation at 480 nm and emission at 535 nm.

### Measurements of glutamine, glucose, ammonia, lactate in the medium

The measurements of these metabolites were performed according to the manual provided by the manufacture of these colorimetric kits. In brief, the cell culture medium was harvested and centrifuged to remove the debris. The medium then was diluted for 2–10 times depending on the applications. The substrates and the enzymes were added to each sample and allow them to react for required time secured from light. Then the colorimetric signals were obtained by reading the plates at assigned wavelength. The detailed manuals are provided by the manufacture (Bioassay Systems). For ammonia measurement, the readings include both ammonia and ammonium.

### Mitochondria inner membrane potential assay

Mitochondrial membrane potential was estimated by staining cells with 5,5′,6,6′-tetrachloro-1,1′,3,3′,-tetra-thylbenzimidazole carbocyanide iodide (JC-1) fluorescence dye (Invitrogen). The cells were treated accordingly. Then the cells were incubated with JC-1 (10 ug/ml) at 37°C for 30 min. Red fluorescence (excitation 550 nm, emission 600 nm) and green fluorescence (excitation 485 nm, emission 535 nm) was detected using a Microplate Reader. The ratio of red to green fluorescence was considered as a degree of mitochondria membrane potential.

### OCR measurement

A Seahorse Bioscience XF24-3 Extracellular Flux Analyzer was used to measure the OCR. MCF-7 cells were seeded in XF 24-well microplates at 2.5 × 10^4^ cells/well. Respiration was measured under different conditions. The procedure followed the standard manual from the manufacture and was described before.^48^

### Immunohistochemistry staining of tissues

Formalin-fixed, paraffin-embedded human normal breast and tumor breast tissue arrays were purchased from Biomax (Rockville, MD, USA) with patients's information available. The procedure of tissue immunohistochemistry analysis for CtBP and SIRT4 was described elsewhere.^19^ In total, there are 50 breast tumors diagnosed as grade II or above and the corresponding normal breast tissues analyzed. The pictures were obtained at the same settings using optical microscopy with a high-resolution camera. The quantitation of antibody staining intensity is through Image J.

### Engrafted tumors in nude mice and GDH activity measurement

The Animal Research Ethics Committee approved all animal procedures. Around 10^4^ cancer cells were used to inject nude mice subcutaneously. When the tumors can be seen to reach ~5 mm diameter in size, the MTOB (750 mg/500 g body weight) was resolved in PBS and was also injected to the subcutaneous space close to the tumors. The MTOB injection was performed three times with 2-day intervals and the control mice were injected with the same volume of PBS at the same position. The tumors were isolated and 5 mg of fresh tumor tissues was used for GDH activity assay and ammonia/ammonium assay. The procedure in analyzing GDH activity followed the manual provided by the manufacture of GDH activity assay kit (Biovision). Protein was also extracted from tissue samples using standard procedure and was analyzed by western blotting.

### TUNEL assay

TUNEL was performed according to the manual provided by the manufacture (Life Technology, now part of Thermo Fisher Scientific).

### Statistical analysis

All the error bars represent the S.D. of the mean from at least three independent biological replicates unless otherwise indicated. Comparisons between two groups were done using unpaired Student's *t* test. *P*<0.05 was considered statistically significant.

## Abbreviations

CtBP: C-terminal-binding protein
TCA cycle: tricarboxylic acid cycle
ChIP: chromatin immunoprecipitation
αKG: α-ketoglutarate
GLS: glutaminase
GDH: glutamate dehydrogenase
NADH: nicotinamide adenine dinucleotide
EMT: epithelial to mesenchymal transition
BPTES: bis-2-(5-phenylacetamido-1,3,4-thiadiazol-2-yl)ethyl sulfide
MTOB: 4-methylthio-2-oxobutyric acid
JC-1: 5,5′,6,6′-tetrachloro-1,1′,3,3′,-tetra-thylbenzimidazole carbocyanide iodide
