# Supplementary material for: CtBP maintains cancer cell growth and metabolic homeostasis via regulating SIRT4
Source: Cell Death Dis. 2015 Jan 29;6(1):e1620–. doi: 10.1038/cddis.2014.587 (PMC4669780; doi:10.1038/cddis.2014.587)
Supplement: Supplementary Information [file cddis2014587x1.doc]

**CtBP maintains cancer cell growth and metabolic homeostasis via regulating SIRT4**

**Running Title: CtBP regulates metabolic homeostasis via SIRT4**

Li Wang^1^, Haisheng Zhou^3^, Yitao Wang^2^, Guozhen Cui^2,4^, Li-jun Di^1^

1. Faculty of Health Sciences, University of Macau, Macau, SAR of People’s Republic of China
2. Institute of Chinese Medical Sciences, University of Macau, Macau, SAR of People’s Republic of China
3. School of life Sciences, Anhui Medical University, Hefei, Anhui Province, People’s Republic of China
4. Bioengineering department, Zunyi Medical college, Zhuhai, Guangdong Province, People’s Republic of China

**Supplementary Figures**

**
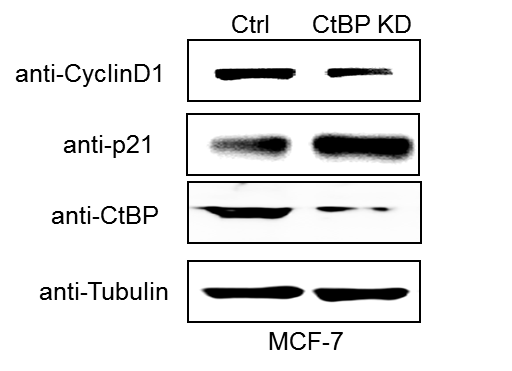
**

**Supplemented Fig1**

Western blotting of *Cyclin D1*, *p21*, *CtBP*, and tubulin in MCF-7 cells without or with *CtBP* knockdown.

**
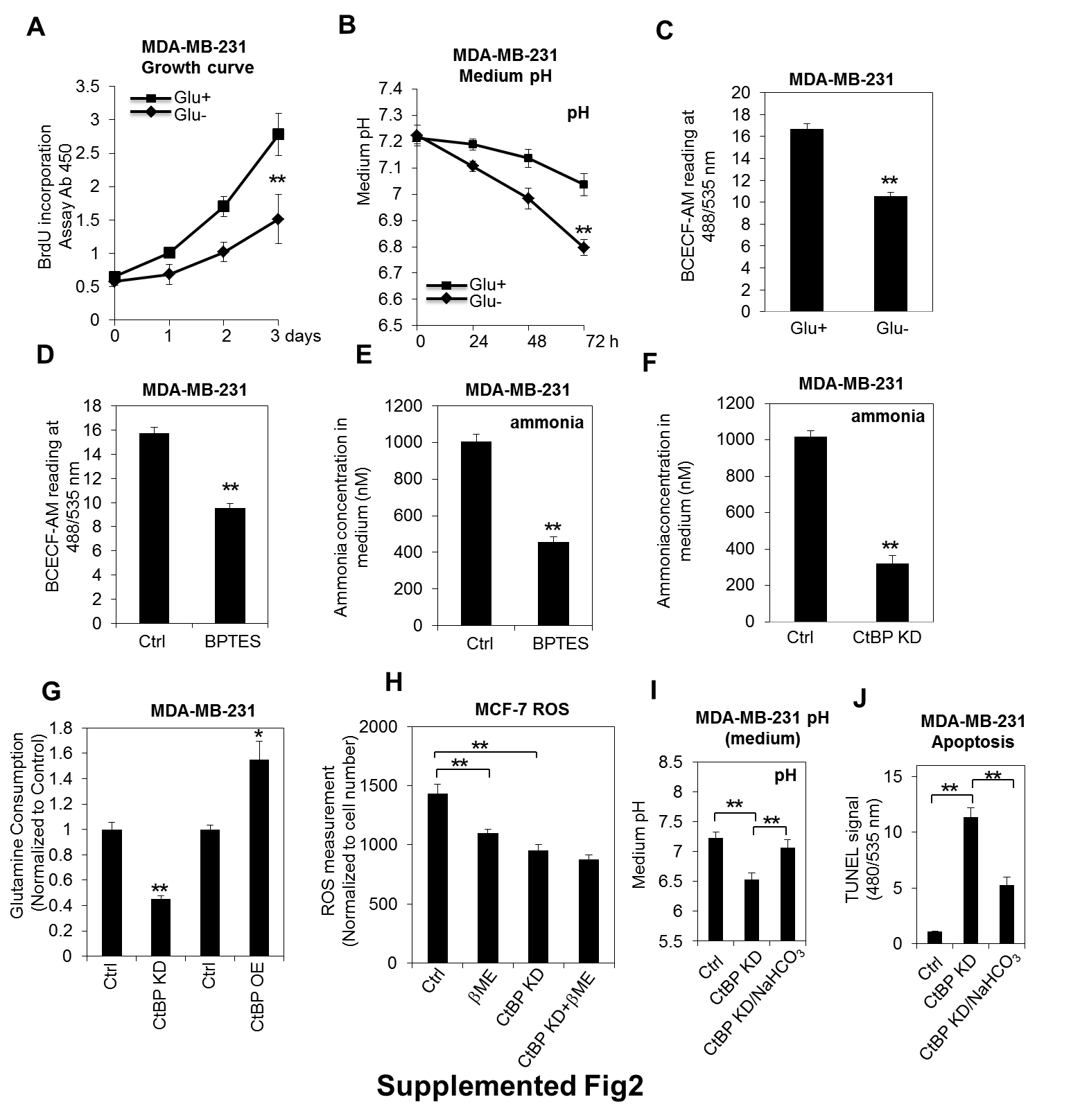
**

**Supplemented Fig2**

A. MDA-MB-231 cell growth curve when glutamine present (Glu+) or absent (Glu-), indicated by BrdU incorporation assay. B. Medium pH was monitored every 24 h for up to 72 h in MDA-MB-231 cells with the conditions of glutamine presence (Glu+) and glutamine absence (Glu-). C. The fluorescence signal of BCECF-AM is negatively correlated with intracellular acidity. Intracellular acidity change upon glutamine withdrawal in MDA-MB-231 cells is shown. D. Intracellular acidity was determined by the reading of BCECF-AM in MDA-MB-231 cells after culture for 24h with BPTES treatment. E. Ammonia production in MDA-MB-231 cells treated by BPTES for 24h. F. Ammonia production in MDA-MB-231 cells without or with CtBP knockdown for 72 h. G. Glutamine consumption in CtBP knockdown (CtBP KD) or over expression (CTBP OE) cells. H. ROS measurement in MCF-7 cells when CtBP was knockdown or the antioxidant βME (100uM) was added. I. Medium pH was measured in the MDA-MB231 cells without or with CtBP knockdown together with NaHCO_3_ treatment. I. MDA-MB-231 cell apoptosis measurement by TUNEL assay with treatment of CtBP knockdown or CtBP knockdown plus NaHCO_3_. The error bars represent the standard deviations (SD) of three independent replicates. * p<0.05 , ** p<0.01.


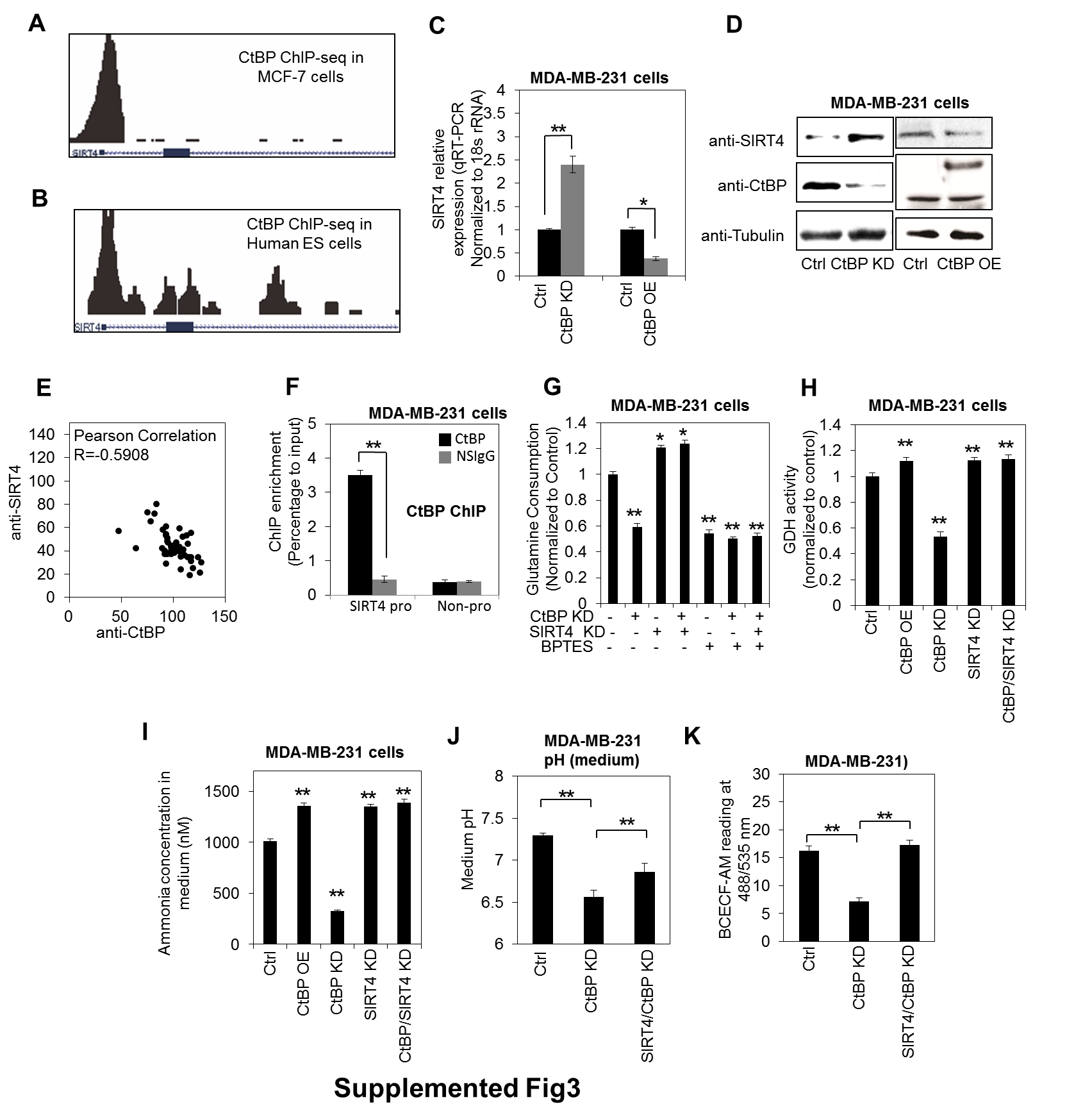


**Supplemented Fig3**

A. CtBP binding peak at SIRT4 promoter in MCF-7 cells. Data is retrieved from GSE36598 published in Di et al 2013 . B. CtBP binding peak in human ES cells available from ENCODE project . C. SIRT4 expression in MDA-MB-231 cells with CtBP knockdown or overexpression. The expression were normalized to 18s rRNA. D. Western blotting of SIRT4 upon CtBP knockdown in MDA-MB-231 cells. CtBP was overexpressed as fusion protein with EGFP. E. Pearson correlation analysis of tissue staining by CtBP and SIRT4. F. CtBP binding at SIRT4 promoter in MDA-MB-231 cells was analyzed by ChIP assay. G. Glutamine consumption was determined in MDA-MB-231 cells with different treatments as indicated. The measurements were performed after 72 hours for gene knockdown and the BPTES dosage was 10uM. H. GDH activity was measured in cells with CtBP overexpression, CtBP knockdown, SIRT4 knockdown or CtBP/SIRT4 double knockdown. I. Ammonia production in MDA-MB-231 cells at the indicated conditions. J and K. Both the medium pH and intracellular acidity are measured in MDA-MB-231 cells with CtBP knockdown and CtBP/SIRT4 double knockdown. The error bars represent the standard deviations (SD) of three independent replicates. * p<0.05 , ** p<0.01. In G, H and I, the p values were calculated between the control sample and the indicated sample respectively.

**Supplemented Fig4**

A. Measurement of intracellular pH in MTOB treated MDA-MB-231 cells. B. CtBP binding at SIRT4 promoter in both MTOB treated and untreated MDA-MB-231 cells. C. SIRT4 expression at mRNA and protein level in MTOB treated and untreated MDA-MB-231 cells. D. Glutamine consumption was measured in MTOB treated and untreated MDA-MB-231 cells. E. GDH activity in MTOB treated and untreated MDA-MB-231 cells. F. Cell viability assay of MDA-MB-231 cells treated by MTOB only or MTOB plus NaHCO3. The error bars represent the standard deviations (SD) of three independent replicates. * p<0.05 , ** p<0.01.

**
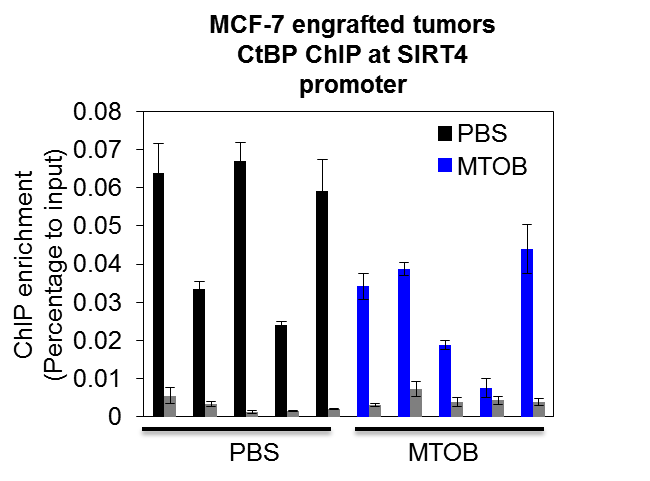
**

**Supplemented Fig5**

ChIP assay of CtBP binding at SIRT4 promoter in engrafted tumors. A non-specific IGG was used for negative control (grey columns) in pull down. The data represent the percentage to input.

**Consortium, E. P., B. E. Bernstein, et al. (2012). "An integrated encyclopedia of DNA elements in the human genome." Nature 489(7414): 57-74.**

**Di, L. J., J. S. Byun, et al. (2013). "Genome-wide profiles of CtBP link metabolism with genome stability and epithelial reprogramming in breast cancer." Nat Commun 4: 1449.**
